# Is the Association between Vitamin D and Cardiovascular Disease Risk Confounded by Obesity? Evidence from the Andhra Pradesh Children and Parents Study (APCAPS)

**DOI:** 10.1371/journal.pone.0129468

**Published:** 2015-06-16

**Authors:** Christopher Paul Baker, Bharati Kulkarni, K. V. Radhakrishna, M. S. Charyulu, John Gregson, Mika Matsuzaki, Amy E. Taylor, Dorairaj Prabhakaran, Raja Sriswan Mamidi, Jonathan Wells, Ian Wilkinson, Carmel McEniery, Yasmin, George Davey Smith, Yoav Ben-Shlomo, Hannah Kuper, Sanjay Kinra

**Affiliations:** 1 Department of Non-Communicable Disease Epidemiology, London School of Hygiene and Tropical Medicine, London, United Kingdom; 2 National Institute of Nutrition, Hyderabad, India; 3 School of Experimental Psychology, University of Bristol, Bristol, United Kingdom; 4 Centre for Chronic Disease Control, New Delhi, India; 5 Childhood Nutrition Research Centre, University College London Institute of Child Health, London, United Kingdom; 6 Division of Experimental Medicine and Immunotherapeutics, University of Cambridge, Cambridge, United Kingdom; 7 School of Social and Community Medicine, University of Bristol, Bristol, United Kingdom; 8 Clinical Research Department, London School of Hygiene and Tropical Medicine, London, United Kingdom; University of Leicester, UNITED KINGDOM

## Abstract

**Background:**

Evidence of an association between serum vitamin D and cardiovascular disease risk is inconsistent and comes predominantly from studies in high-income settings. We assessed the association between serum levels of 25-hydroxyvitamin D_3_ (25(OH)D) and cardiovascular disease risk factors in a population of young Indian adults.

**Methods:**

Cross-sectional analyses of data from APCAPS (Andhra Pradesh Children and Parents Study); a prospective birth cohort study in rural south India. Participants were 1038 (40.3% females) adults aged 18-24 years. Main outcome measures were blood pressures, fasting serum lipids (cholesterols and triglycerides), fasting glucose, insulin, measures of arterial stiffness (aortic augmentation index and aortic pulse wave velocity (aPWV)), carotid intima-media thickness, body mass index (BMI) and body fat (dual X-ray absorptiometry).

**Results:**

Vitamin D deficiency (≤20ng/ml) was observed in 41.1% of this lean (mean BMI: 19.5) and active (mean minutes of moderate or vigorous physical activity per day: 186) population. Vitamin D deficiency was associated with higher median body fat in both males (15.9% body fat in vitamin D deficient males vs. 14.6% in non-deficient males, p<0.05) and females (29.1% body fat in vitamin D deficient females vs. 27.8% in non-deficient females, p<0.05) but no associations were observed between vitamin D deficiency and mean BMI or median fat mass index (FMI). Except a weak inverse association with fasting insulin in males, there was no clear association between serum vitamin D levels and cardiovascular disease risk factors in fully adjusted models.

**Conclusions:**

We did not find clear evidence for an association between serum vitamin D levels and cardiovascular disease risk factors. Our results, consistent with the limited evidence from randomised trials of vitamin D supplementation and Mendelian randomisation experiments, suggest that the postulated link between serum vitamin D and cardiovascular disease may be non-causal. Instead, it may be attributable to confounding by lifestyle factors such as obesity and physical inactivity which may provide more fruitful targets for cardiovascular disease prevention.

## Introduction

Systematic reviews of observational studies have reported inverse associations between serum vitamin D levels and a range of cardiovascular disease risk factors, most notably hypertension, but also dyslipidaemia, abnormal glucose homeostasis and atherosclerosis [1–6]. However, the few randomised trials of vitamin D supplementation completed to date have failed to show a clear beneficial effect on cardiovascular events, raising questions about the causal role of vitamin D in cardiovascular disease (CVD) [7, 8].

Observational studies examining the association between vitamin D levels and CVD risk have predominantly been conducted in high-income countries, where the perceived rise in the prevalence of vitamin D deficiency has taken place alongside a rise in the prevalence of obesity and physical inactivity[8]. Obesity and physical inactivity are both independent risk factors of CVD and are also associated with lower levels of vitamin D, thus suggesting a non-causal explanation for their association [9]. The hypothesis that observational evidence for an association between vitamin D and cardiovascular risk may be confounded is partially supported by evidence from Mendelian Randomisation (MR) studies. These studies, which have found no clear association between genetic markers of vitamin D and cardiovascular risk or cardiovascular mortality, provide evidence of a causal association between genetic markers of obesity and circulating vitamin D levels [10–13].

Some observational studies have tried to control for obesity when assessing the relationship between vitamin D and CVD, using body mass index (BMI) as a proxy, and found inconsistent results; however, BMI is non-specific and residual confounding cannot be ruled out [14, 15]. A more robust approach to control for imprecisely measured confounders, like obesity and physical inactivity, is to conduct the study in a population with an absent or extremely low prevalence of the putative confounder. We examined a lean population of young adults from rural South India in order to study the relationship between 25(OH)D and a comprehensive range of cardiovascular risk factors. The availability of data on body fat assessed by Dual energy X-ray Absorptiometry (DXA) and physical activity, lacking in many previous studies, further allowed us to reliably assess the potential confounding role of these key risk factors of CVD.

## Methods

### Study Design and Population

The Andhra Pradesh Children and Parents Study (APCAPS) is a prospective cohort study conducted in 29 villages near Hyderabad city in South India. It was established through the long-term follow-up of family members of participants of the Hyderabad Nutrition Trial (HNT) (1987–1990), in which supplemental nutrition was offered to pregnant women and young children from a block of 15 villages (an intervention arm) [16, 17]. We conducted cross-sectional analysis of data from the second wave of this study when serum vitamin D and cardiovascular risk factors were assessed in the index trial children (those born between 1987 and 1990 and reliably linked during initial follow up in 2003–5).

### Ethics Statement

The study received approval from the ethics committees of the National Institute of Nutrition (NIN) (Hyderabad, India) and London School of Hygiene and Tropical Medicine (London, UK). Approval was also sought from the Indian Council for Medical Research and the village heads and their committees in each of the study villages. Written informed consent or witnessed thumbprint if illiterate was obtained from the participants prior to their inclusion in the study.

### Measurements

The study participants were interviewed and examined by trained interviewers at the National Institute of Nutrition (NIN) in Hyderabad between 2009 and 2010.

#### Questionnaire data

Socio-demographic and lifestyle information was collected through a structured questionnaire. Socio-economic status was measured with a subset of questions (14/29) from the Standard of Living Index (SLI), a household level asset-based scale devised for Indian settings [18].

Physical activity undertaken in the week preceding the clinic was assessed using a questionnaire developed and evaluated previously in a similar setting [19]. The frequency of and average length of time spent on activities were documented for multiple domains, including work, travel, leisure (sports/games/exercise), household chores, and time spent being sedentary.

#### Physical examination

Height was measured to the nearest 1mm with a portable plastic stadiometer (Leicester height measure; Chasmors, London) and weight was measured to the nearest 0.1kg with a digital SECA balance. Each measure was taken twice and the average was used for analysis.

Lean mass and fat mass were measured with Dual energy X-Ray Absorptiometry (DXA). 95% of scans were performed on a Hologic Discovery A model and 5% on a Hologic 4500W. The same software version was used on both machines. The whole body scan was performed with participants supine on the scanning bed. Women suspected of pregnancy (n = 22) were excluded from DXA scanning. Standard Hologic software options were used to define regions of the body (head, arms, trunk, and legs). Scans were coded for artefacts by visual inspection; incomplete scans and those with major movement were excluded. DXA abdominal fat measures showed excellent agreement with abdominal adipose tissue measured by magnetic resonance imaging (MRI) in a subset of the study population [20]. Fat mass index, considered to be a more valid measure of body composition[21], was calculated as body fat mass (kg) / height (m)^2^ and lean mass index (LMI) was calculated as lean mass (kg) / height (m)^2^.

Systolic and diastolic blood pressure was measured with a validated oscillometric device (Omron M5-I, Matsusaka Co., Japan) in the supine position, with appropriate cuff sizes. Each measure was taken three times and the average of the second and third reading was used for analysis.

Carotid intima-media thickness was measured on the right-hand side near wall of the distal common carotid artery using ultrasound. Images were analysed using Carotid Plaque Texture Analysis software (LifeQ, Engomi, Cyprus). Images were first normalised to enable reproducibility of texture features and plaque types. Where more than one image was available for a participant an average of all image readings was used for analysis.

Aortic augmentation index, a measure of arterial wave reflections, was measured by applanation tonometry on the radial artery using the SphygmoCor device (AtCor Medical Pty Ltd, West Ryde, Australia). If the first two readings were not within 5% points of each other, a third was taken. The mean of the two closest readings was used for analysis. Aortic pulse wave velocity, a measure of arterial stiffness, was determined using the Vicorder device (Skidmore Medical, Bristol, UK). Blood pressure cuffs were placed around the upper thigh and around the neck at the level of the carotid artery to measure femoral and carotid pulses. The cuffs were then inflated to 60mmHg and waveforms recorded using a volume displacement method [22].

#### Laboratory assessment of blood samples

Participants attended morning clinics and were asked to fast overnight. The time of their last meal was recorded. Venous blood samples (20mL) were collected. All assays, except for fasting glucose and serum vitamin D (which were measured at NIN), were performed in the Genetics and Biochemistry Laboratory of the South Asia Network for Chronic Disease, New Delhi. 25(OH)D was measured using a high-performance liquid chromatography (HPLC) method and detected at 265nm using an ultraviolet detector (CV: 7%). Serum High-Density Lipoprotein cholesterol (HDL) was estimated directly by an elimination method, total cholesterol by an enzymatic endpoint method, and triglycerides by GPO-PAP method. Low-Density Lipoprotein cholesterol (LDL) was calculated using the Friedewald formula (total cholesterol—HDL cholesterol—triglycerides) [23]. Insulin was measured with the enzyme-linked immunosorbent assay (ELISA) method, using solid phase, two-site enzyme immunoassay (MERCODIA, Uppsala, Sweden). Fasting glucose was measured using an oxidase-peroxidase system [24].

The quality of assays was checked with regular external standards and internal duplicate assays and monitored by All India Institute of Medical Sciences (AIIMS). The Cardiac Biochemistry Lab, AIIMS, is part of the UK National External Quality Assessment (www.ukneqas.org.uk) programme and External Quality Assessment Scheme from RANDOX for quality assurance of insulin and biochemical assays respectively.

#### Statistical Analysis

Socio-demographic and biological characteristics of the study sample were compared by vitamin D status. Normally distributed continuous variables were reported as means (± standard deviation) and skewed continuous variables were reported as medians (with interquartile range). Comparison of means and medians used unpaired t-tests and Mann-Whitney tests respectively. Chi-squared tests were used to compare categorical variables.

Exploratory analysis highlighted linear relationships between 25(OH)D and most CVD outcomes. The exposure variable (25(OH)D) and two outcome variables (triglycerides and insulin) were log-transformed to achieve a distribution closer to normal.

Associations between 25(OH)D and cardiovascular risk factors were examined using linear mixed-effects regression models. To account for possible clustering of exposures, robust standard errors were applied at the household and village levels. Two models, stratified by sex, were fitted and adjusted for potential confounders or intermediaries: model 1—age and intervention status; model 2—as in model 1, plus SLI, occupation, smoking status, time spent in moderate or vigorous physical activity, and body fat. Analysis of 25(OH)D level by month of test suggested that the season in which the clinical examination took place influenced levels of serum vitamin D. Month of test was therefore also included as possible confounder in model 2.

All statistical analysis was carried out using Stata version 13 (StataCorp, College Station, Texas).

## Results

All 2581 living members of the Hyderabad Nutrition Trial cohort (born between 1987 and 1990) were invited to participate; 1446 (56%) attended. Attendees were more likely to be male than were non-attendees (68% vs 28%). Due to machine malfunction, assessment of 25(OH)D was conducted on blood samples of 1038 (72%) participants. Those without 25(OH)D data were more likely to be male as compared to those with (89% vs 60%), but comparisons of lifestyle characteristics illustrated similarities between groups (Table 1). After excluding those who did not fast overnight (n = 36) 1002 participants were left for analysis of fasting glucose, insulin, HDL and LDL.

**Table 1:** Key characteristics of APCAPS participants with a serum vitamin D result (n = 1038) compared to APCAPS participants without a serum vitamin D result (n = 408); 2009–10.

	Males			Females		
	With a serum vitamin D result (n = 620)	Without a serum vitamin D result (n = 361)	p-value	With a serum vitamin D result (n = 418)	Without a serum vitamin D result (n = 47)	p-value
Age (years)	20.99	20.43	<0.001	21.01	20.93	0.63
Body Mass Index (kg/m^2^)	19.76	19.63	0.49	19.12	19.19	0.87
Body fat (%)	15.95	15.2	0.02	28.35	27.5	0.33
Standard of Living Index	36.68	36.67	0.84	34.73	34.55	0.89

Values are means. P-values are based on unpaired t-tests for heterogeneity in means, with appropriate degrees of freedom

Table 2 shows the distribution of characteristics for the study sample by vitamin D status. The mean age of the sample was 21 (SD: 1.1) and the mean BMI was 19.5 (SD: 2.9). Forty one percent (52% of females, 34% of males) of the participants were vitamin D deficient (25(OH)D≤20ng/ml [25]). Participants with vitamin D deficiency had higher levels of % body fat, but did not have higher BMI or FMI, as compared to those without deficiency. Males with vitamin D deficiency were less physically active compared to non-deficient males, but no such difference was noted in females.

**Table 2:** Distribution of socio-demographic and biological characteristics of APCAPS participants (n = 1038), 2009–10.

	Males (n = 620)				Females (n = 418)			
	n	Vitamin D Deficient* (n = 211)	Vitamin D non-Deficient (n = 409)	p-value†	n	Vitamin D Deficient* (n = 216)	Vitamin D non-Deficient (n = 202)	p-value†
**Socio-demographics**								
Age (years)	620	21.1(1.1)	20.9(1.1)	0.16	418	21.0(1.2)	21.0(1.2)	0.56
Standard of Living Index	614	18.6(4.2)	18.7(4.1)	0.91	415	18.1(4.4)	17.1(4.5)	0.02
Occupation (%)¶	619			0.55	418			0.09
At home/ unemployed		3.8	2.9			33.8	41.6	
Student		39.3	35.1			35.2	24.8	
Manual		52.1	55.4			27.8	31.7	
Professional		4.7	6.6			3.2	2.0	
Smoking (%)§	619			<0.01	417			-
Never		93.4	83.6			100	100	
Former/Current		6.6	16.4			0	0	
MVPA Time (mins/day)‡	620	176.0(106.4 to 265.7)	201.4(122.3 to 311.0)	0.05	418	68.4(35.6 to 117.8)	71.1(32.4 to 162.9)	0.87
**Biological characteristics**								
Body Mass Index (kg/m2)	619	19.75(2.66)	19.76(2.82)	0.95	418	19.12(3.1)	19.11(2.78)	0.99
Body Fat (%)‡	620	15.9(12.9 to 19.4)	14.6(11.8 to 18.9)	0.02	380	29.1(25 to 33)	27.8(23.9 to 31.7)	0.04
Fat Mass Index (body fat kg/ m2) ‡	619	3.03(2.29 to 4.11)	2.71(2.14 to 3.83)	0.06	380	5.32(4.31 to 6.71)	5.01(4.11 to 6.30)	0.21
Lean Mass Index (lean mass kg/ m2)	619	15.6(1.6)	15.7(1.6)	0.54	381	12.7(1.7)	12.9(1.3)	0.24
Systolic blood pressure (mmHg)	620	117.1(9.5)	117.6(9.0)	0.55	418	109.9(10.0)	108.6(8.3)	0.15
Diastolic blood pressure (mmHg)	620	70.4(8.4)	70.1(7.5)	0.59	418	69.0(8.7)	67.8(7.4)	0.15
Carotid IMT (mm)	601	0.6(0.1)	0.6(0.1)	0.83	407	0.5(0.1)	0.5(0.1)	0.40
Aortic Augmentation Index (%)	615	2.7(10.0)	1.5(11.2)	0.20	408	3.4(13.4)	2.2(12.1)	0.33
Aortic Pulse Wave Velocity (m s-1)	620	5.97(0.04)	5.99(0.03)	0.71	409	5.81(0.04)	5.74(0.04)	0.25
Insulin (mU/L)‡ ф	601	3.8(2.8 to 5.2)	3.8(2.8 to 5.1)	0.91	401	4.1(2.9 to 5.8)	3.6(2.4 to 5.2)	0.02
Fasting glucose (mmol/L) ф	601	4.8(0.5)	4.8(0.5)	0.13	401	4.8(0.4)	4.8(1.0)	0.94
Triglycerides (mg/dl)‡	620	86(70 to 110)	94(74 to 118)	0.02	418	83(65 to 107.5)	85.5(68 to 117)	0.09
Total cholesterol (mmol/L)	620	4.00(0.93)	4.03(0.9)	0.76	418	3.98(0.85)	4.07(0.9)	0.31
HDL cholesterol (mmol/L) ф	601	1.0(0.2)	1.0(0.2)	0.74	401	1.1(0.2)	1.1(0.2)	0.49
LDL cholesterol (mmol/L) ф	598	2.5(0.9)	2.5(0.8)	0.60	401	2.4(0.7)	2.5(0.7)	0.97
25(OH)D (ng/ml)‡	620	15.2(12.1 to 17.7)	29(24.5 to 35.9)	-	418	15(11.7 to 17.6)	24.25(22.2 to 29)	-

All values are means(SD) unless otherwise stated.

MVPA = Moderate or Vigorous Physical Activity; 25(OH)D = 25-hydroxyvitamin D_3_, IMT = Intima-Media Thickness, HDL = High-density lipoprotein, LDL = Low-density lipoprotein

* We used a cut-off of ≤20ng/ml to define deficiency (equal to 50nmol/l) [18].

^†^ p-values are based on unpaired t-tests for heterogeneity in means, with appropriate degrees of freedom.

^‡^ non-normal distribution; median (inter-quartile range) presented, and p-values are based on Mann-Whitney rank-sum tests for equality.

^Ф^ Analysis of fasting glucose, insulin, HDL cholesterol and LDL cholesterol exclude participants who did not fast (n = 36)

^§^ Smoking status; former user = ceased use >6 months ago; current user = used in the last 6 months.

^¶^ Manual occupations include roles such as labourers, craftsmen, servants, postal staff and farmers; professional occupations include role such as teachers, accountants, clinicians, business owners and engineers.

Although we saw a weak inverse association with fasting serum insulin (in males only), there were no consistent associations between 25(OH)D and other CVD risk factors in unadjusted or fully adjusted models (Table 3 for males and Table 4 for females). The association between 25(OH)D and fasting insulin was not attenuated on adjustment for body fat, despite the association between insulin and body fat. We explored the possibility of effect modification of this association by body fat, but found no strong evidence for this (P interaction = 0.19). Regarding physiological CVD risk factors, % body fat displayed a strong inverse association with serum vitamin D in both sexes but for FMI a similar association was only observed in females. Diastolic blood pressure appeared to have a small inverse association with vitamin D in females, but this attenuated after adjustment.

**Table 3:** Association of serum vitamin D (25(OH)D)† with cardiovascular risk factors in a sample of young Indian males from the Andhra Pradesh Children and Parents Study (n = 620)Ф; 2009–10.

	Model 1			Model 2		
	n	β coefficient(95% CI)	p	N	β coefficient(95% CI)	P
**Outcomes—Physiological**						
Body Mass Index (kg/m^2^)*	619	0.07(-0.43 to 0.57)	0.77	613	0.00(-0.50 to 0.51)	1.00
Body Fat by DXA (%)*	620	-0.06(-0.11 to -0.00)	0.03	614	-0.05(-0.1 to 0.00)	0.06
Fat Mass Index (body fat kg/ m^2^)*	619	-0.05(-0.12 to 0.02)	0.15	613	-0.05(-0.13 to0.02)	0.16
Systolic BP (mmHg)	620	-0.05(-1.68 to 1.58)	0.95	614	0.55(-1.10 to 2.20)	0.52
Diastolic BP (mmHg)	620	-0.60(-1.98 to 0.77)	0.39	614	-0.06(-1.39 to 1.27)	0.93
Carotid IMT (mm)	601	0.01(-0.01 to 0.03)	0.43	595	0.00(-0.02 to 0.02)	0.89
Aortic Augmentation Index (%)	615	-0.58(-2.51 to 1.35)	0.56	609	-0.93(-2.81 to 0.96)	0.33
aPWV (m s^-1^)	620	-0.00(-0.11 to 0.11)	0.94	614	0.01(-0.10 to 0.12)	0.82
**Outcomes—Biochemical**						
Insulin (mU/L)†	599	-0.11(-0.21 to -0.02)	0.02	593	-0.12(-0.21 to -0.02)	0.01
Fasting Glucose (mmol/L)	601	-0.07(-0.16 to 0.02)	0.14	595	-0.09(-0.18 to 0.01)	0.08
Triglycerides (mg/dl)†	620	0.02(-0.04 to 0.09)	0.50	614	0.03(-0.03 to 0.09)	0.89
Cholesterol (mmol/L)	620	0.07(-0.10 to 0.23)	0.42	614	0.16(0.00 to 0.31)	0.05
HDL Cholesterol (mmol/L)	601	-0.01(-0.04 to 0.03)	0.64	595	0.00(-0.04 to 0.03)	0.93
LDL Cholesterol (mmol/L)	598	0.07(-0.07 to 0.21)	0.34	592	0.13(-0.01 to 0.27)	0.07

25(OH)D = 25-hydroxyvitamin D3; DXA = Dual X-ray Absorptiometry; BP = Blood Pressure; IMT = Intima-Media Thickness; aPWV = Aortic Pulse Wave Velocity; HDL = High-density lipoprotein; LDL = Low-density lipoprotein

Model 1 adjusts for age and intervention status. Model 2, as in Model 1, plus further adjustment for lifestyle factors (standard of living index, occupation, time spent in moderate or vigorous physical activity, smoking status), body fat and month of test. Results are based on linear mixed effect regression models with robust standard errors to account for clustering at the household and village level, rounded to 2 decimal places.

* Model 2 excludes body fat

^Ф^ Analysis of fasting glucose, insulin, HDL cholesterol and LDL cholesterol exclude participants who did not fast (n = 36)

^†^Logged values

**Table 4:** Association of serum vitamin D (25(OH)D)† with cardiovascular risk factors in a sample of young Indian females from the Andhra Pradesh Children and Parents Study (n = 418)Ф; 2009–10.

	Model 1			Model 2		
	N	β coefficient(95% CI)	P	N	β coefficient(95% CI)	P
**Outcomes—Physiological**						
Body Mass Index (kg/m^2^)*	418	-0.56(-1.22 to 0.11)	0.10	375	-0.61(-1.29 to 0.07)	0.08
Body Fat by DXA (%)*	380	-0.07(-0.11 to -0.02)	0.004	375	-0.05(-0.1 to -0.01)	0.02
Fat Mass Index (body fat kg/m^2^)*	380	-0.10(-0.17 to -0.02)	0.01	375	-0.09(-0.16 to-0.01)	0.02
Systolic BP (mmHg)	418	-1.49(-3.59 to 0.61)	0.16	375	-1.16(-3.44 to 1.12)	0.32
Diastolic BP (mmHg)	418	-2.60(-4.47 to -0.73)	0.01	375	-1.84(-3.74 to 0.06)	0.06
Carotid IMT (mm)	407	-0.01(-0.03 to 0.01)	0.18	364	-0.02(-0.04 to 0.00)	0.05
Aortic Augmentation Index (%)	408	-0.47(-3.41 to 2.47)	0.75	366	-1.31(-4.44 to 1.82)	0.41
aPWV (m s^-1^)	409	-0.09(-0.22 to 0.04)	0.16	374	-0.01(-0.15 to 0.13)	0.90
**Outcomes—Biochemical**						
Insulin (mU/L)†	391	-0.14(-0.28 to -0.01)	0.04	356	-0.02(-0.16 to 0.12)	0.77
Fasting Glucose (mmol/L)	401	-0.01(-0.19 to 0.16)	0.87	365	0.08(-0.1 to 0.27)	0.38
Triglycerides (mg/dl)†	418	0.09(0.01 to 0.17)	0.03	375	0.04(-0.05 to 0.13)	0.36
Cholesterol (mmol/L)	418	0.16(-0.04 to 0.35)	0.12	375	0.10(-0.1 to 0.31)	0.31
HDL Cholesterol (mmol/L)	401	0.02(-0.03 to 0.07)	0.35	365	0.01(-0.04 to 0.06)	0.81
LDL Cholesterol (mmol/L)	401	0.07(-0.11 to 0.24)	0.46	365	0.06(-0.12 to 0.24)	0.50

25(OH)D = 25-hydroxyvitamin D3; DXA = Dual X-ray Absorptiometry; BP = Blood Pressure; IMT = Intima-Media Thickness; aPWV = Aortic Pulse Wave Velocity; HDL = High-density lipoprotein; LDL = Low-density lipoprotein

Model 1 adjusts for age and intervention status. Model 2, as in Model 1, plus further adjustment for lifestyle factors (standard of living index, occupation, time spent in moderate or vigorous physical activity, smoking status), body fat and month of test. Results are based on linear mixed effect regression models with robust standard errors to account for clustering at the household and village level, rounded to 2 decimal places.

* Model 2 excludes body fat

^Ф^ Analysis of fasting glucose, insulin, HDL cholesterol and LDL cholesterol exclude participants who did not fast (n = 36)

^†^Logged values

## Discussion

In this community-based sample of lean and active young adults from rural India we found no clear association between serum vitamin D levels and CVD risk factors, including blood pressures, arterial stiffness, carotid intima-media thickness and fasting lipids, glucose and insulin. Serum vitamin D was inversely associated with DXA-assessed body fat percentage in both sexes and with FMI in females only, but no relationship was observed between serum vitamin D and BMI.

### Comparison with previous research

The high prevalence of vitamin D deficiency (41%) in our study was similar to estimates from other studies of healthy populations in India and other settings [26–30]. The lack of any robust associations between 25(OH)D and a range of CVD risk factors in our study contradicts findings from existing observational studies [4, 7]. However, this is in line with limited evidence from intervention studies of vitamin D supplementation and MR experiments using genetic markers of vitamin D deficiency, which have also failed to detect any clear associations between the two.

As expected, BMI, and DXA-assessed % body fat and FMI were all strongly correlated. However, these three measures did not display similar associations with serum vitamin D. Our finding of an association between serum vitamin D and DXA-assessed body fat in both sexes, and FMI in females, partly supports our hypothesis that the reported association between vitamin D levels and CVD risk, despite adjustment for BMI, may reflect residual confounding from inaccurate measurement of body fat. This is supported by evidence from studies that also had DXA-assessed body fat measurements [31–34].

Our study also corroborates the previous finding that people who do more moderate or vigorous physical activity have higher levels of serum vitamin D [35, 36]. This may simply reflect greater exposure to UVB sunlight that such activity entails. Manual labour (including agricultural work) is common in this rural setting and exercise for leisure is mostly in the form of outdoor sport. Similarly, female participants more commonly reported house-work which would reduce their exposure to UVB sunlight; suggesting a possible explanation for their higher prevalence of vitamin D deficiency. A low level of serum vitamin D may still be considered a marker of an unhealthy lifestyle, notably physical inactivity and obesity, rather than being causally related to cardiovascular disease risk [37, 38].

### Strengths and limitations

The current study is one of the first observational studies to examine the association between 25(OH)D and CVD risk in a population exhibiting a very low prevalence of obesity or physical inactivity. We also had a sufficiently large sample and access to high quality measurements of the exposure of interest, a wide range of outcomes and key confounding variables. In particular, our assessment of physical activity was very detailed. We lacked data on skin colour which may have been useful given the influence of skin pigmentation on synthesis of vitamin D [39]. The participants were required to attend a clinic 1–2 hours away from home which limited our response rate, particularly among females and those in full-time employment, and could have resulted in selection bias, although no major baseline differences between participants and non-participants were noted [12]. Our analyses were cross-sectional and therefore limit the ability to infer causality. Our population displayed a relatively low risk of CVD therefore generalisation of our results to populations with higher risk of CVD may not be possible. Given the progressive emergence of CVD risk over the life-course, we cannot rule out the possible role of serum vitamin D in CVD outcomes later in life, which may not have been captured in this young population.

### Public health implications

Our data, albeit limited to a particular population, do not support a causal role for vitamin D in CVD risk. Given the inconsistent evidence on this subject to date, further research is needed before vitamin D can be recommended for prevention of CVD. Maintenance of optimal vitamin D levels may still be desirable for other outcomes (e.g. bone health[40]). For now, efforts to prevent CVD should remain focussed on established risk factors, such as obesity and physical inactivity.
